# A Genome-Wide Screen of CREB Occupancy Identifies the RhoA Inhibitors Par6C and Rnd3 as Regulators of BDNF-Induced Synaptogenesis

**DOI:** 10.1371/journal.pone.0064658

**Published:** 2013-06-06

**Authors:** Adam Lesiak, Carl Pelz, Hideaki Ando, Mingyan Zhu, Monika Davare, Talley J. Lambert, Katelin F. Hansen, Karl Obrietan, Suzanne M. Appleyard, Soren Impey, Gary A. Wayman

**Affiliations:** 1 Department of Veterinary and Comparative Anatomy, Pharmacology and Physiology, Program in Neuroscience, Washington State University, Pullman, Washington, United States of America; 2 Vollum Institute, Oregon Health and Science University, Portland, Oregon, United States of America; 3 Oregon Stem Cell Center, Oregon Health and Science University, Portland, Oregon, United States of America; 4 Department of Pediatrics, Oregon Health and Science University, Portland, Oregon, United States of America; 5 Department of Neuroscience, Ohio State University, Columbus, Ohio, United States of America; University of Louisville, United States of America

## Abstract

Neurotrophin-regulated gene expression is believed to play a key role in long-term changes in synaptic structure and the formation of dendritic spines. Brain-derived neurotrophic factor (BDNF) has been shown to induce increases in dendritic spine formation, and this process is thought to function in part by stimulating CREB-dependent transcriptional changes. To identify CREB-regulated genes linked to BDNF-induced synaptogenesis, we profiled transcriptional occupancy of CREB in hippocampal neurons. Interestingly, de novo motif analysis of hippocampal ChIP-Seq data identified a non-canonical CRE motif (TGGCG) that was enriched at CREB target regions and conferred CREB-responsiveness. Because cytoskeletal remodeling is an essential element of the formation of dendritic spines, within our screens we focused our attention on genes previously identified as inhibitors of RhoA GTPase. Bioinformatic analyses identified dozens of candidate CREB target genes known to regulate synaptic architecture and function. We showed that two of these, the RhoA inhibitors Par6C (Pard6A) and Rnd3 (RhoE), are BDNF-induced CREB-regulated genes. Interestingly, CREB occupied a cluster of non-canonical CRE motifs in the Rnd3 promoter region. Lastly, we show that BDNF-stimulated synaptogenesis requires the expression of Par6C and Rnd3, and that overexpression of either protein is sufficient to increase synaptogenesis. Thus, we propose that BDNF can regulate formation of functional synapses by increasing the expression of the RhoA inhibitors, Par6C and Rnd3. This study shows that genome-wide analyses of CREB target genes can facilitate the discovery of new regulators of synaptogenesis.

## Introduction

Most excitatory synapses in the mammalian brain are found on small, actin-rich protrusions of the dendritic membrane known as dendritic spines [1]–[4]. Functional and structural changes at spines and synapses are believed to be the basis of learning and memory in the brain [1]–[7]. Abnormal spine formation is highly correlated with a variety of mental disorders, including schizophrenia, mental retardation, Down’s syndrome, and autism spectrum disorders [8]–[14]. Dendritic spine formation requires precise cytoskeletal regulation, and many of the key proteins regulating this process are members of the Rho-family of small GTPases [15]–[18]. Activation of Rac1 or CDC-42 is thought to stimulate the formation of dendritic spines, while RhoA activation during early neuronal development generally inhibits synaptic development [19]–[25].

Long-term changes in spine morphogenesis often depend on de novo gene expression [26], [27]. In particular, activation of CREB-dependent transcription has been linked to *de novo* and developmental synaptogenesis [25], [28], [29]. Neurotrophic factors, such as brain derived neurotrophic factor (BDNF), are both activators of CREB-dependent transcription and regulators of synaptogenesis [30]–[39]. In hippocampal neurons, BDNF activation of the TrkB receptor regulates CREB-dependent gene expression largely by activating the ERK-dependent kinase signaling cascade, resulting in direct phosphorylation of CREB Ser133 by Msk1/2 [40]–[42]. Previous studies have identified molecules, such as miR132, that are expressed in a CREB-dependent manner following BDNF-treatment [43], [44]. The effect of increased miR132 expression is implicated in regulation of the actin cytoskeleton, and it promotes changes in synaptic connectivity and stimulates dendritic spine formation [20], [25], [40]. Therefore, we sought to identify additional CREB-regulated genes that contribute to BDNF-mediated synapse formation.

To achieve this goal, we utilized chromatin immunoprecipitation (ChIP) and next generation sequencing to identify CREB-target sites in hippocampal neurons. Interestingly, bioinformatic analyses identified an alternate, non-canonical CRE motif that was highly enriched at CREB targeted genes, facilitated recruitment of CREB, and was sufficient for CREB-regulated transcription. Modeling of the CREB bZip-CRE crystal structure showed that this variant CRE maintained the same interactions as the canonical motif. Gene ontology analysis to select putative CREB-targets that regulate the actin cytoskeleton resulted in the identification of two CREB- and BDNF-regulated molecules known to inhibit RhoA, Par6C (Pard6a) and Rnd3 (RhoE). Both Rnd3 and Par6C have been reported to inhibit RhoA signaling via activation of p190RhoGAP, and thus play a potential role in BDNF-dependent spine formation [23], [40], [45].

We demonstrate that BDNF-induced CREB-dependent synaptogenesis requires the expression of the RhoA inhibitors, Rnd3 and Par6C. Moreover, analyses of CREB ChIP-Seq data identified an alternate, non-canonical, CRE motif that is occupied by CREB in the Rnd3 promoter, and is sufficient to confer CREB responsiveness. These discoveries shed insight into the processes by which CREB and neurotrophins regulate synapse formation and synaptic remodeling.

## Methods

### Reagents, Plasmids, and Primers

The following reagents were purchased from the indicated sources: Recombinant human BDNF (Peprotech), U0126 (Calbiochem). pCAG-ACREB [41], [46], and caCREB [43], [47] plasmids have been described previously. Rnd3(RhoE) [27], [31], [36], [48]–[51] construct was previously described and given as a gift from Dr. Anne Ridley. Myc-Par6C and myc-p190GAP were PCR cloned into pCAGGS from Rat cDNA using standard methods. For each target gene, three short hairpin RNAs (shRNAs) targeting a 19–22 nucleotide target sequence were designed using shRNA design tool at RNAi Central (http://cancan.cshl.edu/RNAi_central/main2.cgi). Serial Cloner (http://serialbasics.free.fr/Serial_Cloner.html) was used to design two complementary oligos incorporating the target sequence and a short hairpin sequence (TTCAAGAGA) surrounded by BglII and HindIII restriction sequences. The complementary oligos were annealed and cloned into either the pSUPER or the pSUPER GFP vector (Oligoengine) between the BglII and the HindII sites. Each sh-RNA was tested for effectiveness of knockdown in both HEK-293 cells and hippocampal neurons. The most effective sh-RNA was then used throughout the described studies. Exogenous knockdown by sh/si-RNAs was used based on detection limitations of endogenous Par6C and Rnd3 expression using Rnd3 and Par6C antibodies, as well as transfection efficiency limitations in cultures. Luciferase Reporter Constructs: The canonical CRE (CRE: 5′ agcttggctcatgacgtagtaagca, 3′ gatctgcttactacgtcatgagcca) and the novel CRE (novel: 5′ agcttggctcatggcgtagtaagca, 3′ gatctgcttactacgccatgagcca) were phosphorylated and ligated into the BglII and XhoI sites of dephosphorylated pGL3 basic (promega).

Antibodies: anti-pErk (rabbit, Cell signaling), anti-Erk1/2 (mouse Santa Cruz), anti-pCREB (mouse, Chemicon), anti-c-Myc (mouse, Sigma), anti-Rnd3 (mouse, Millipore), anti-Par6 (rabbit, Sigma), anti-Synapsin1 (mouse, Synaptic systems), anti-VGlut1 (mouse, Neuromab). Primers used for ChIp-Seq analyses, and cloning primers provided in (Table S1).

### Cell Culture

Animal use for hippocampal cultures was carried out in compliance with Washington State University IACUC approved protocols ASAF 0317-011 and ASAF 04020-003. These protocols were specifically approved by Washington State University s institutional IACUC review board. Hippocampal neurons (3.0×10^4^ cells per square centimeter) were cultured from P1–2 Sprague–Dawley rats on plates coated with poly-L-lysine (Sigma; molecular weight 300,000) as described previously [52]–[54]. Hippocampal neurons were maintained in Neurobasal A media (Invitrogen) supplemented with B27 (Invitrogen), 0.5 mM L-glutamine, and 5 µM cytosine-D-arabinofuranoside (Sigma; added at 2 DIV). Hippocampal neurons used for chromatin immunoprecipitation assays, ChIP-Seq, qPCR, and Western blotting experiments all were treated on DIV6 as written in text and figure legends. For BDNF or UO126 treatment, reagent was first diluted prior to being added to native in-well media to reach the final in-well target concentration. Hippocampal neurons used for dendritic spine analysis and electrophysiological analysis were transfected with various constructs on DIV6 and then treated on DIV7± BDNF in media to a final concentration of 50 ng/mL BDNF. Cells treated without BDNF received the same amount of media at this time. On DIV12, cells were either fixed or used for electrophysiological recordings.

### Transfection

Primary hippocampal neurons were transfected with LipofectAMINE 2000 (Invitrogen). For transfection of 24 wells of a 24 well plate, 50 µL of Lipofectamine 2000 was added to 2.5 mL of NBA and incubated for 5 minutes. DNA plasmids for each of the treatments were mixed in separate tubes, with enough total plasmid DNA for 1 µg/well (ex. mRFP-βActin used at 5% of total plasmid). Native media in wells were collected and kept warm, and 500 µL of warm GM was placed in the well. NBA/L2K mix was added to DNA mixtures for 20 minutes, before 100uL/well of DNA/NBA/L2K mix was added to each well for 30 minutes. After 30 minutes, media was aspirated, and 500 µL of warm native media was returned to well. In each experiment, we optimized DNA amounts to minimize toxicity and maximize transfection efficiency. Lipofectamine 2000 transfection efficiency was 0.5–5%.

### Slice Culture and Transfection

Organotypic hippocampal slices from P5 Sprague–Dawley rats were cultured as described previously [20], [47], [54]. To visualize spine morphology, we transfected slices with pCAG-TFP (Tomato) using a Helios Gene Gun (BioRad), according to the manufacturer's protocol on DIV2. Following transfection, slices were allowed to recover for 48 h before the slice culture media was exchanged for fresh media on DIV4. During this exchange of slice culture media, BDNF treated hippocampal slices received slice culture media with 50 ng/mL BDNF. On DIV6 slices were fixed (see below), mounted, and imaged using a confocal microscope. Dendritic spine density and morphology were measured as described below.

### Chromatin Immunoprecipitation and ChIP-Seq

ChIP was conducted as described previously [40], [55]. Briefly, hippocampal neurons were fixed with 1% formaldehyde for 10 min at room temperature. Chromatin was sonicated and immunoprecipitated using indicated antibodies overnight. All ChIP-PCR analysis was conducted using real-time PCR. Table S1 contains the primers used for ChIP-Seq analyses. ChIP-Seq libraries were generated and sequenced using standard Illumina protocols. 25 bp reads were mapped to the mouse genome (UCSC mm9) using the Bowtie2 algorithm using trimmed 40 bp reads and allowing for 3 mismatches. Only sequences that mapped to a single genomic location were used. ChIP-Seq areas of enrichment at an FDR of 0.001 were determined using a 500 bp sliding-window approach based on a previous algorithm [56]. A small number of annotated satellite repeat artifacts were manually removed. ____ChIP-Seq data has been submitted to GEO and can be accessed at:.

### RNA Isolation, Reverse Transcription and Quantitative Real-time PCR

Total or nuclear RNA was isolated using the Trizol (Invitrogen) method following manufacturer’s protocol, and reverse-transcribed using MMLV (Invitrogen). Real time PCR was conducted as described previously [40]. All RT-PCR data utilized standard curve real-time PCR. Primers are provided in Table S1.

### Bioinformatic and Statistical Analyses

ChIP-Seq areas of enrichment at an FDR of 0.001 were determined using a custom 500 bp sliding-window approach based on a previous algorithm [56], but implemented in C++. Input genomic DNA was sequenced, analyzed using the same sliding window algorithm, and a 2-fold cut off was used to remove “background” peaks. Similar results were seen with Cis-Genome, another algorithm that incorporates genomic input background correction [57].

ChIP-Seq data was annotated and visualized using R. Annotation was downloaded from the UCSC genome browser (mm9). Profile plots of ChIP-Seq data relative to RefSeq genes were generated in R by removing redundant RefSeq gene annotation, setting all gene lengths equal to 1, and plotting median-adjusted tag density. Statistical comparisons between ChIP-Seq data sets and genomic data were conducted in R and utilized Monte-carlo analyses and the Wilcoxon rank-sum test for significance testing. Both ChIP genomic input reads and randomized genomic extents were used to create Monte-carlo background models with equivalent results. *De novo* motif analyses utilized 500 bp regions flanking the sequence tag center of mass of the top 1000 ChIP-Seq peaks with and without the canonical CRE (ranked by total sequence counts). The Weeder and YMF algorithms produced similar results using flags that allowed for the presence of more than one 6- mer motif on both strands [1], [3]. Statistical analyses and visualization of motif data utilized custom R and C++ scripts and all analyses. Gene ontology analysis was conducted using DAVID [8] and p-values was adjusted using the Storey q-test [15]. Comparison of low-throughput biological data utilized the Student’s t-test or ANOVA followed by a post-test where appropriate.

### Quantification of Spine Density and Morphology

Hippocampal neurons were transfected with mRFP-βactin±test plasmids or oligos as indicated. Expression of fluorescently tagged actin allows visualization of dendritic spine density, size and shape. Expression of low levels of βactin has no significant effect on either spine density or size or shape [19], [20], and similar spine density changes were seen with the same manipulations in organotypic slice cultures transfected with TFP. Neurons were transfected on DIV 6, then fixed using PHEM/PFA (4% paraformaldehyde, 60 mM PIPES, 25 mM HEPES, 5 mM EGTA, 1 mM MgCl2, pH7.4) on DIV 12 for 20 min at room temperature, washed with PBS then mounted using Elvanol mounting buffer. Fluorescent images were obtained with Slidebook 4.2 Digital Microscopy Software driving an Olympus IX81 inverted confocal microscope (Olympus Optical, Tokyo, Japan) with a 60× oil immersion lens, numerical aperture 1.4, and resolution 0.280 µm. Dendritic spine density was measured on primary and secondary dendrites at a distance of at least 150 µm from the soma. Two to three segments of dendrites from at least 24 neurons (more than 36 for most conditions) were analyzed for each data point reported. Each experiment was repeated at least twice (three times or more for most conditions) using independent culture preparations. Dendrite length was determined using ImageJ 1.41 (National Institutes of Health, Bethesda, MD) and the neurite tracing program Neuron J [26]. Spines were manually counted and classified. Spines were categorized using slightly modified criteria as described in [28]. Mushroom: Dendritic protrusion with a distinct actin rich head wider than the diameter of the shaft (Thin spines from Harris et al. criteria were included in this distinction). Stubby: Actin rich protrusion with a head size similar to the total length of the spine and no discernible shaft. Filopodia: Protrusion without a discernible actin rich spine head. Total spines were the combined total of Mushroom and Stubby spine types. Dendritic spine head width was measured on 50–100 spines on 10–12 neurons per condition in two independent experiments using ImageJ 1.41, measuring the widest diameter of an actin-rich spine head.

### Immunocytochemistry

Transfected neurons were treated and fixed as described above. After fixation, cells were rinsed in PBS and permeabilized with 0.1% Triton X-100 detergent (Bio-Rad Laboratories, Hercules, CA), followed by two rinses in PBS, and blocked with 8% bovine serum albumin (Serological Corp., Norcross, GA) in PBS for 1 h. Cells were again rinsed with PBS, followed by a 24-h incubation period with anti-VGLUT1 (Synaptic Systems, Goettingen, Germany) or anti-synapsin1 (Synaptic Systems) diluted in 1% BSA, following the manufacturer's protocol, at 4°C. Then, cells were rinsed thrice with PBS, incubated in Alexa Fluor 488 goat-anti-mouse IgG in 1% BSA following the manufacturer's protocol (Invitrogen) for 2 h at room temperature, rinsed again with PBS, and mounted with elvanol. Imaging was performed as described above. Spines were determined to be co-localized if any portion of the spine head overlapped with VGLUT1 or Synapsin1 puncta (50–100 spines on 10–12 neurons were analyzed for each condition in two separate experiments).

### Western Blotting

To analyze Par6C and Rnd3 expression, cultures were treated as specified in figure legends, and each well was lysed in 50 µL RIPA buffer (Upstate) with Phosphatase Inhibitor II and III (Sigma) and Protease Inhibitor (Roche). Samples were then dounce homogenized and spun in a microcentrifuge to pellet debris, and frozen at −80°C for storage. Samples were prepared using NuPage LDS Sample Buffer (Invitrogen) with 0.5 M DTT and heated at 70°C for 10 minutes before equal volumes were loaded into NuPage 4–12% Bis-Tris gels (Invitrogen) and run using NuPage Mops Buffer. Protein was transferred to PVDF using Tris-Glycine buffer. Blots were blocked using Aquablock (East Coast Biotech) for 1 hour, and then probed with anti-Par6C (Sigma, c-terminal), anti-Rnd3 (Upstate), and anti-ERK ½ (Loading control)(Santa Cruz) diluted in Aquablock overnight. Blots were then washed with PBS before application of Rockland IR secondary antibodies (anti-RB 700 and anti-MS 800) for 2 hours. Finally, blots were washed with PBS again before blots were scanned using Li-COR infra-red Odyssey Scanner to image blots. Band intensities were measured using ImageJ and normalized to ERK2 band intensities.

### Whole-Cell Recordings, mEPSC Analysis

Patch-clamp experiments were performed on cultured hippocampal neurons transfected with mRFP-β-actin-transfected and test plasmids ± BDNF pretreatment. Recordings were made on DIV12 to DIV14. The culture medium was exchanged by an extracellular solution containing 140 mM NaCl, 2.5 mM KCl, 1 mM MgCl_2_, 3 mM CaCl_2_, 25 mM glucose, and 5 mM HEPES; pH was adjusted to 7.3 with KOH, and osmolality was adjusted to 310 mOsM. Cultures were allowed to equilibrate in a recording chamber mounted on an inverted microscope (IX-71; Olympus Optical) for 30 min before recording. Transfected cells were visualized with fluorescence (Olympus Optical). Recording pipettes were pulled (P-97 Flaming/Brown micropipette puller; Sutter Instrument Company, Novato, CA) from standard-wall borosilicate glass without filament (o.d. = 1.5 mm; Sutter Instrument Company). The pipette-to-bath d.c. resistance of patch electrodes ranged from 4.0 to 5.2 MΩ, and they were filled with an internal solution of the following composition: 25 mM CsCl, 100 mM CsCH_3_O_3_S, 10 mM phosphocreatine, 0.4 mM EGTA, 10 mM HEPES, 2 mM MgCl_2_, 0.4 mM Mg-ATP, and 0.04 mM Na-GTP; pH was adjusted to 7.2 with CsOH, and osmolality was adjusted to 296 to 300 mOsM. Miniature EPSCs (mEPSCs) were isolated pharmacologically by blocking GABA receptor chloride channels with picrotoxin (100 µM; Sigma-Aldrich), blocking glycine receptors with strychnine (1 µM; Sigma-Aldrich), and blocking action potential generation with tetrodotoxin (500 nM; Tocris Bioscience, Ellisville, MO). Recordings were obtained using a Multiclamp 700B amplifier (Molecular Devices, Sunnyvale, CA). Analog signals were low-pass Bessel-filtered at 2 kHz, digitized at 10 kHz through a Digidata 1440A interface (Molecular Devices), and stored in a computer using Clampex 10.2 software (Molecular Devices). The membrane potential was held at −70 mV at room temperature (25°C) during a period of 0.5 to 2 h after removal of the culture from the incubator. Liquid junction potentials were not corrected. Data analysis was performed using Clampfit 10.2 software (Molecular Devices) and Mini-Analysis 6.0 software (Synaptosoft, Decatur, GA). The criteria for a successful recording included an electrical resistance of the seal between the outside surface of the recording pipette and the attached cell >2 GΩ and neuron input resistance >240 MΩ. A 5 min. recording time was used for analysis of the mEPSCs.

## Results

### ChIP-Seq Analysis of CREB Occupancy in Synaptically Active Hippocampal Neurons

**Figure 1:**
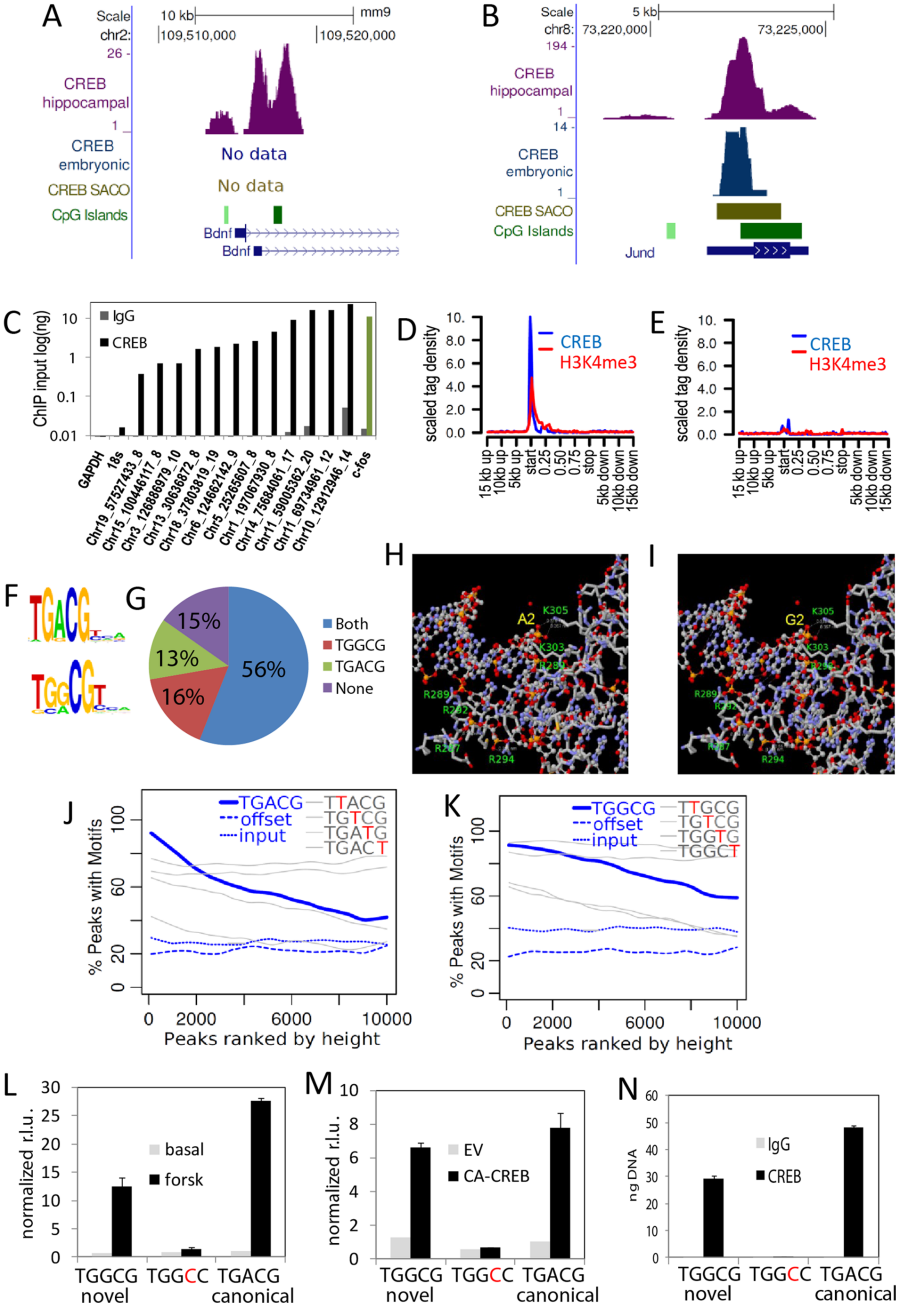
CREB ChIP-Seq identifies non-canonical CRE motif. (A and B) UCSC Genome browser tracks depicting CREB ChIP-Seq peaks and CRE motifs at known targets BDNF and JunD. (C) Hippocampal neuron CREB ChIP material was interrogated by real-time PCR using primers designed to a region within ∼200 bp of the indicated peak centroid (weighted average of positions). All ChIP-Seq peaks were confirmed to be occupied by CREB (n = 3, SEM). 18s and GAPDH are negative controls while c-fos (green bar) is a positive control. (D and E) ChIP-Seq tag density relative to the top third of expressed genes in hippocampal neurons (D) and the bottom third (E). Expression levels were determined by sorting hippocampal RNA-Seq data by normalized tag counts within RefSeq gene exons. All Ref-Seq gene lengths were scaled to 1. (F) *De novo* motif algorithms identify canonical and non-canonical motif in CREB ChIP-Seq data. (G) Pie chart depicts presence of CRE and non-canonical motif within 2kb of a ChIP-Seq peak. (H and I) Visualization of electrostatic interactions between DNA phosphate backbone and CREB bZIP domain for the consensus somatostatin CRE (H) and a modeled TGGCG mutated CRE (I). Charged amino acids making phosphate contacts are labeled. Carbon atoms are white, oxygen atoms are red, nitrogen atoms are blue and phosphate atoms are yellow. (J and K) Accumulation plots depicting the percentage of indicated motifs present in all CREB ChIP-Seq peaks (500 bp window surrounding center of mass). Offset denotes a control region 2 kb upstream of ChIP-Seq loci. Random indicates randomized motif positions. The grey lines depict motifs in which a single base has been changed to T. (L and M) Hippocampal neurons were transfected with the indicated luciferase reporter constructs and treated with forskolin (L) or co-transfected with constitutively active CREB (ca-CREB) (M). (N) PC12 cells were transfected with the indicated reporter constructs and subjected to ChIP for CREB. Primers directed against the reporter enhancer site were used to assess recruitment of CREB via real-time PCR.

Activation of CREB-dependent transcription has been shown to increase synaptogenesis [30]. Nevertheless, the complement of genes that contribute to CREB-regulated synaptogenesis are largely uncharacterized. To identify CREB target sites in synaptically active neurons, we performed high-throughput sequencing of CREB ChIP material derived from mature cultured hippocampal neurons (Table S2). Approximately 12 million reads were generated and peak calling identified ∼33,000 regions (FDR of 0.001%) containing ∼7 million reads. The antibody used was previously shown to be selective for CREB and was extensively validated for ChIP [40]. As expected, repeat CREB ChIP at a random subset of peaks confirmed CREB occupancy at all tested regions (Figure 1C). CREB ChIP-Seq peaks were markedly enriched at the 5′ end of known genes (Figure 1A, 1B, and 1D) and 29% were within 1 kb of an annotated RefSeq transcriptional start site (Table S2). We also generated ChIP-Seq data for H3K4me4, a mark associated with transcriptional start sites. CREB occupancy was highly correlated with both active gene expression and trimethylated histone 3 (H3K4me3) (Figure 1D and E; data not shown). The enrichment for CREB at gene 5′ gene ends is consistent with previous ChIP-Sequencing data from our lab [43]. Moreover, as previously observed [40], a substantial percentage of CREB peaks were located at inter- or intra-genic regions distal to annotated transcriptional start sites (Table S2).

### Bioinformatic Comparison with Previous CREB ChIP-sequencing Data

To further validate our hippocampal neuron ChIP-Seq data we mapped our earlier CREB ChIP-SACO [40] data set to the mouse genome resulting in 4,338 predicted CREB binding sites. Interestingly, ∼75% of CREB SACO regions were within 2 kb of a hippocampal ChIP-Seq peak (Wilcoxon rank sum, p<1×10^−11^) (Table S3) suggesting substantial overlap for CREB occupancy between cell types. Interestingly, our hippocampal data predicted CREB occupancy at many genes that are expressed in hippocampal neurons but not PC12 cells, including BDNF and Homer1 (Table S3). A recently published forebrain embryonic neuron CREB ChIP-Seq data set also showed a high-degree of overlap with our data but contained only 1,623 peak calls (63% of embryonic neuron peaks were within 2kb of a hippocampal peak from our data; p<1×10^−10^). To better examine any overlap between the two data sets, we re-mapped and re-analyzed the embryonic neuron ChIP-Seq reads with our pipeline to obtain 4,783 regions of predicted CREB enrichment (FDR of 0.001%). 59% of these embryonic neuron ChIP-Seq peaks (∼2,600) were within 2 kb of a hippocampal neuron ChIP-Seq peak (Wilcoxon rank sum, p<1×10^−10^, Table S3). Importantly, randomized (5.4%) or genomic input controls (5.8%, Wilcoxon rank sum, p = 0.59) showed markedly less overlap. Interestingly, we identified ∼29,000 predicted CREB binding sites that were not detected in the much smaller forebrain embryonic neuron data set. Importantly, these candidate regulatory sites were correlated with transcriptional start sites and canonical CREB response elements (CREs) to approximately the same extent as CREB sites detected in both data sets (data not shown). Moreover, our analyses have identified ∼3,000 CREB-bound loci identified by 3 distinct ChIP-Sequencing studies (Table S3) as well as tens of thousands of new potential CREB-regulatory regions (Table S2).

### Identification of an Alternate, Non-canonical, CREB-responsive Element

A puzzling observation from earlier studies was that many predicted CREB target regions were not adjacent to canonical CREB responsive elements (CREs). Although some of these anomalous interactions may represent indirect binding events or lack of spatial specificity, many highly ranked loci were not adjacent to a canonical CREB CRE. *De novo* motif analysis of the top 500 hippocampal CREB ChIP-Seq peaks that do not contain a canonical motif identified an alternate, non-canonical, motif (TGGCG) (Figure 1F) that was highly enriched at CREB ChIP-Seq peaks (Wilcoxon rank sum, p<1×10^−11^). Interestingly, ∼72% of CREB peaks were within 2 kb of a non-canonical motif and ∼69% were adjacent to the canonical CRE (TGACG) (Figure 1G, Database 1). Although the palindromic CRE was the first consensus sequence proposed, subsequent studies have shown that a half-site CRE can drive CREB-dependent transcriptional activation and is found in many prototypical CREB-responsive genes (for review see [58]). 56% of ChIP-Seq peaks were within 2 kb of both a canonical and a non-canonical motif (while only 2% of randomized regions were adjacent to both motifs. Importantly, introduction of the novel motif G to A mutation into the published structure of the CREB bZIP bound to the canonical CRE revealed no change in hydrogen bond distances indicating that this motif is compatible with this structural model of CREB interaction (Figure 1H and I). [46]. To assess whether the new motif is a genuine CRE, we assessed its accumulation in a 1kb window flanking CREB ChIP-Seq peaks rank-ordered by sequence tag content. Interestingly, both the non-canonical and canonical motif showed similar levels of accumulation at hippocampal neuron ChIP-Seq peaks (Figure 1J and 1K) and forebrain embryonic neuron ChIP-Seq peaks (Table S3). Importantly, regions selected from randomized “genomic input” or regions 2 kb distal to the peaks did not show rank-ordered motif enrichment. Introduction of *in silico* “T” mutations generated similar reductions in enrichment for both the novel and the canonical motifs. Thus, the non-canonical motif shows similar levels of enrichment at predicted CREB target sites and predicts thousands of novel direct targets in both our ChIP-Seq data and earlier data sets (Figure 1F, (Table S2, Table S3)).

We next tested whether the non-canonical motif was sufficient to drive CREB-dependent transcription. Introduction of the motif into a heterologous reporter was sufficient to drive cyclic AMP- and CREB-dependent transcription (Figure 1L and M) and a single nucleotide substitution attenuated this regulation. Moreover, CREB was selectively recruited to this motif as assessed by chromatin immunoprecipitation of the transfected reporter (Figure 1N) or by biotinylated CRE oligo pull down (data not shown).

### The Non-canonical CRE is Associated with CREB Occupancy

**Figure 2:**
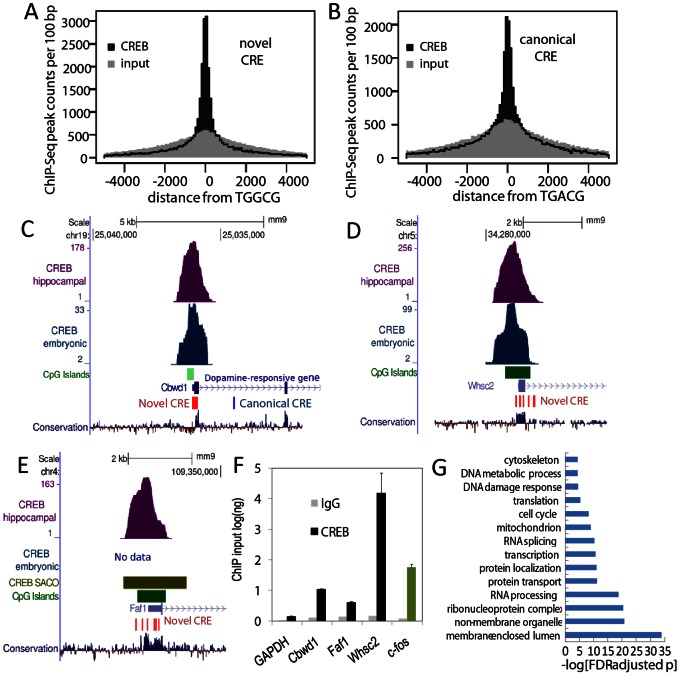
The non-canonical CRE is associated with CREB occupancy and CREB-responsiveness of endogenous genes. (A and B) Histograms depict spatial accumulation of mouse hippocampal CREB ChIP-Seq peaks relative to all genomic occurrences of the canonical CRE (TGACG) and the non-canonical CRE (TGGCG). Randomized peaks over the same genomic extent are depicted in grey. (C-E) UCSC genome browser tracks show hippocampal CREB ChIP-Seq (CREB hip), embryonic cortical neuron CREB ChIP-Seq (CREB embryonic) data relative to RefSeq genes, non-canonical CRE motifs (red), and CpG islands (green). Rat CREB SACO data mapped to the mouse genome is also depicted with purple bars denoting SACO cluster extent (CREB SACO). The depicted loci did not contain the canonical CRE motif. (F) Chromatin from rat and mouse hippocampal neurons was immunoprecipitated with the indicated antibodies. Real-time PCR with primers directed against the ChIP-Seq peak (mouse) or the orthologous rat locus (rat) were used to assess CREB occupancy (n = 3; SEM). Significance was assessed using the Storey-adjusted Fisher exact test (FDR-adjusted p). (G) All RefSeq genes whose annotated transcriptional start is within 1 kb of a hippocampal neuron ChiP-Seq peak were selected for gene ontology analyses. Significance was assessed using the Storey-adjusted Fisher exact test (FDR-adjusted p).

If the non-canonical CRE contributes to recruitment of CREB it should show similar spatial correlation with CREB occupancy as the canonical motif. A histogram of CREB occupancy at all genomic positions of the non-canonical motif shows the same narrow spatial localization as the known motif (Figure *2*A and B). Moreover, enrichment of ChIP-Seq peak counts at both motifs was highly significant (Wilcoxon rank-sum: p<1×10^−11^). Similar results were seen with both embryonic ChIP-Seq data [43], and PC12 SACO data (data not shown). To test whether this was due to promoter or GC bias we selected ChIP-Seq peaks that were greater than 5 kb distal to annotated promoters and performed a similar analysis. Enrichment for both motifs in this smaller set of non-GC-rich regions was also very significant (Wilcoxon rank-sum: p<1×10^−6^) (data not shown). To test whether this motif is correlated with functional regulation of gene expression, we selected ChIP-Seq peaks adjacent to proximal promoters that contained the non-canonical, but not the canonical motif (1000 bp window). In this data the distribution of the non-canonical motif at individual genes showed tight correlation with hippocampal ChIP-Seq peaks, and with ChIP data from other studies (Figure 2C-E). ChIP-Seq PCR analyses confirmed significant CREB occupancy at these regions (Figure 2F). These data suggest that the non-canonical motif can recruit CREB to target genes *in vivo.*

### Identification of the RhoA Inhibitors Par6C and Rnd3 as BDNF- and CREB-regulated Genes

**Figure 3:**
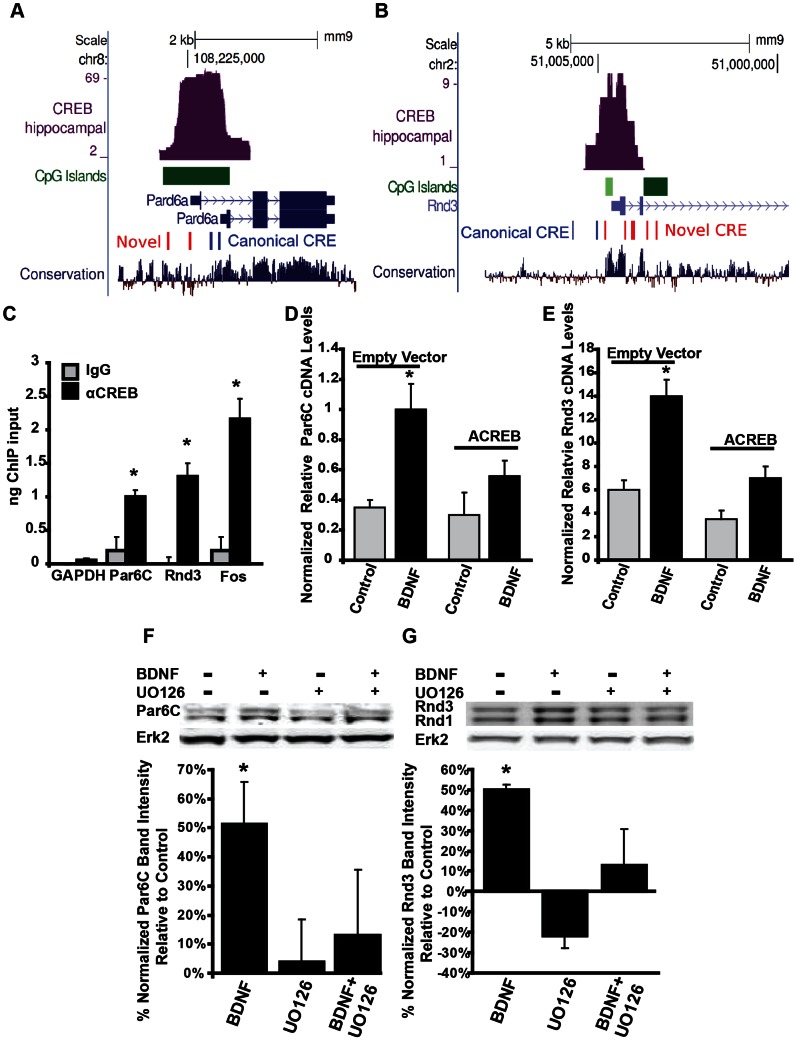
The RhoA inhibitors Rnd3 and Par6C are BDNF-regulated CREB target genes. (A and B) UCSC Genome browser tracks depicting CREB ChIP-Seq peaks and CRE motifs at the Par6C/pard6a (A) and Rnd3 (B) genes. The CRE motifs in the Par6C gene are the canonical motif (TGACG) while all three of the CRE motifs in the Rnd3 gene are the non-canonical CRE (TGGCG). (C) Mouse hippocampal neuron chromatin was immunoprecipitated with the indicated antibodies. Primers to the Fos, Par6C, and Rnd3 ChIP-Seq peaks were used to assess occupancy of CREB at these loci. GAPDH represents a control locus not predicted to be occupied by CREB. (IgG, n = 2; CREB, n = 4; SEM). (D and E) Hippocampal neurons were transfected with empty vector (E.V.) or ACREB DNAs, and treated on DIV6±50 ng/mL BDNF for 1 or 2 hrs. RNA was reverse transcribed and relative cDNA levels of Par6C (D) and Rnd3 (E) were assessed by real-time PCR. Relative cDNA levels were normalized to relative GAPDH cDNA levels also measured by real time PCR. (n = 4; SEM). (F–G) DIV6 cultured hippocampal neurons were treated ±50 ng/mL BDNF for 4 hours ±1 hour pre-treatment with 20 µM U0126, lysed, and then analyzed by Western Blot for Par6C and Rnd3 with ERK2 used as a loading control. Changes in protein band intensity were determined by densitometry using image J. Graph depicts percent change in band intensity relative to control, and normalized to ERK2 loading control band intensity, representing 3 separate experiments. Data is presented as % of control ± SEM. (± SEM, Statistical analyses utilized ANOVA and Tukey’s post-test, *p<0.01 compared to control).

Gene ontology analysis of genes adjacent to our CREB ChIP-Seq peaks showed enrichment for cytoskeletal genes and genes involved in protein transport and localization (Figure 2G). In particular, the RhoA-signaling pathway has been shown to effect synapse formation. Therefore, we selected the known RhoA-inhibitors Par6C and Rnd3 as candidate genes from the set of cytoskeletal regulating genes detected in our gene ontology analysis. The Par6C ChIP-Seq locus was associated with a promoter-proximal CpG island, a conserved canonical cAMP response element (Figure 3A), and was selectively immunoprecipitated by CREB antisera (Figure 3C). A control region in the GAPDH promoter was not detected. Interestingly, the Rnd3 promoter region contained a cluster of non-canonical CREs that precisely overlap the ChIP-Seq peak and were conserved in mammals (Figure 3B, data not shown). The closest canonical CRE (∼1 kb distal) was not conserved and did not show significant CREB recruitment in repeat ChIP-PCR assays (data not shown). The Rnd3 locus was selectively immunoprecipated with CREB antisera supporting the idea that the non-canonical CRE mediates CREB recruitment (Figure 3C). We next tested whether the expression of these genes was regulated by CREB. BDNF-treatment triggered a rapid rise in Par6C and Rnd3 mRNA levels (Figure 3D and E). The ability of BDNF to stimulate Par6C and Rnd3 expression was markedly attenuated by expression of ACREB, a potent inhibitor of CREB binding (Figure 3D and E). We next examined whether BDNF stimulated an increase in the expression of Par6C and Rnd3 protein expression. Hippocampal neurons were stimulated with BDNF, U0126 (a Mek inhibitor), or a combination of U0126 and BDNF for four hours and cell extracts were analyzed by western blot for Par6C and Rnd3. BDNF increased the expression of both Par6C and Rnd3 by approximately 50% and this increase was reduced by pretreatment with the Mek inhibitor, U0126 (Figure 3F–G). These results indicate that the promoters for both Par6C and Rnd3 are occupied by CREB in vivo and that activation of the BDNF-Mek/Erk-CREB pathway increases Par6C and Rnd3 expression.

### BDNF-dependent Spinogenesis Requires CREB Activity

**Figure 4:**
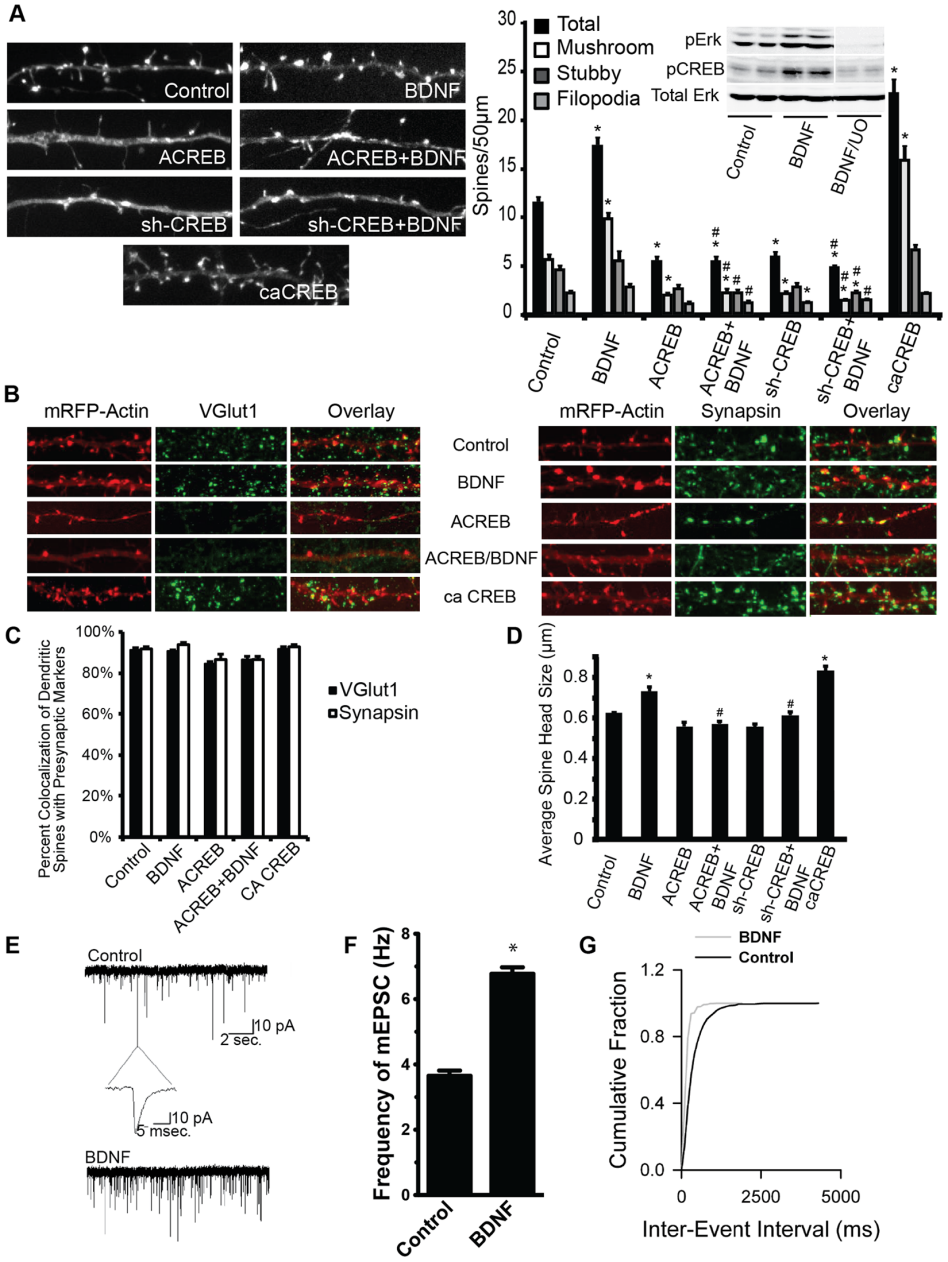
CREB is activated by BDNF-stimulation, and required for BDNF-dependent spinogenesis. DIV6 cultured hippocampal neurons were transfected with m-RFP-βActin ± empty vector (Control and BDNF), ± ACREB, ± sh-CREB, or ± caCREB, and then treated ±50 ng/mL BDNF from DIV7–12. Cultures were then used for electrophysiological recordings, or fixed, mounted, immunostained, and imaged. A) Representative images and quantification of dendritic spine type and filopodia density is shown, with total spine number as the combination of mushroom and stubby spines. Inset panels) DIV6 cultured hippocampal neurons were treated ±50 ng/mL BDNF for 20 minutes ±1 hour pre-treatment with 20 µM U0–126, lysed and then analyzed by Western Blot, using anti-phospho-ERK, anti-phospho-CREB, and anti-ERK2 (loading control) antibodies. B) Representative images of neurons immunostained using anti-VGlut1 and anti-Syanapsin1 antibodies. C) Quantification of percent co-localization of presynaptic markers with dendritic spine heads (50–100 spines measured on 10–12 hippocampal neurons per condition in two experiments). D) Quantification of average spine head width (50–100 spines measured on 10–12 neurons per condition in two experiments). E) Representative traces of mEPSCs recorded from control or BDNF treated neurons at DIV12 following ± BDNF stimulation from DIV7–12 (50–80 neurons in 6 experiments). F) Average frequency of mEPSCs. G) mEPSC cumulative distribution of inter-event intervals. (± SEM, Statistical analyses utilized Student’s t-test and ANOVA with Tukey’s post-test, *p<0.001 compared to control, #p<0.001 compared to BDNF).

BDNF is known to stimulate synaptogenesis in both hippocampal and cortical neurons, and BDNF has been shown to activate CREB-dependent transcription in neurons [27], [31], [36], [49]–[51]. Consistent with previous studies, we show that CREB function is required for spine formation (Figure 4A). Treatment of hippocampal neurons with BDNF activates the Mek/Erk and CREB pathways (Figure 4A, inset panel). Pre-treatment with the Mek inhibitor U0126, blocks BDNF-mediated activation of Erk and CREB, and confirms that CREB is downstream of TrkB and the Mek/Erk pathways. To investigate the role of CREB in BDNF-stimulated spinogenesis, we inhibited CREB activity by expressing ACREB [52], or by knocking down endogenous CREB using an sh-RNA construct. To activate CREB-dependent transcription we expressed caCREB (constitutively active CREB, CREB_DIEDML_) [47]. Expression of either ACREB or sh-CREB did not significantly affect neuronal health or alter gross dendritic morphology in neurons grown in the presence of B27 ([55] and data not shown). Treatment of cultured hippocampal neurons with BDNF stimulated an increase in “mature” mushroom-shaped dendritic spines (Figure 4A). The increase in mushroom shaped dendritic spines by BDNF was largely attenuated by transfection with either ACREB or sh-CREB. ACREB expression decreased the density of all spine types below basal levels, and sh-CREB expression, with or without BDNF, decreased all spine types below control levels (Figure 4A). Transfection with constitutively active CREB (caCREB), on the other hand, greatly increased dendritic spine density of mushroom spines well above control levels (Figure 4A). Since the size of the dendritic spine head is related to the strength of the synapse [2], [4], [18], the width of dendritic spine heads were also measured. BDNF-treatment significantly increased average spine head width, and this increase was phenocopied by expression of caCREB (Figure 4D). Expression of either ACREB or sh-CREB had no significant effect on basal spine size, and both constructs blocked BDNF-induced increases in spine size (Figure 4D). To determine whether the observed BDNF-induced increases in dendritic spines represent areas of functional synaptic connectivity, we assessed the co-localization of dendritic spine heads with presynaptic markers. Approximately 80–90% of dendritic spines showed co-localization with VGLUT1 (glutamatergic presynaptic maker) and Synapsin I (general presynaptic marker) puncta (Figure 4B and C). To address whether BDNF induced changes in the number of functionally active synapses in our system we recorded miniature excitatory post-synaptic currents (mEPSCs) from dissociated hippocampal neurons. Treatment of hippocampal neurons with BDNF increased mEPSC frequency approximately 2-fold (Figure 4E–G), but did not significantly effect rise/decay time or mEPSC amplitude (data not shown). These data suggest that CREB-dependent transcription is necessary for the BDNF-induced formation of functional dendritic spines.

### RhoA Activity Inhibits BDNF-induced Spinogenesis

**Figure 5:**
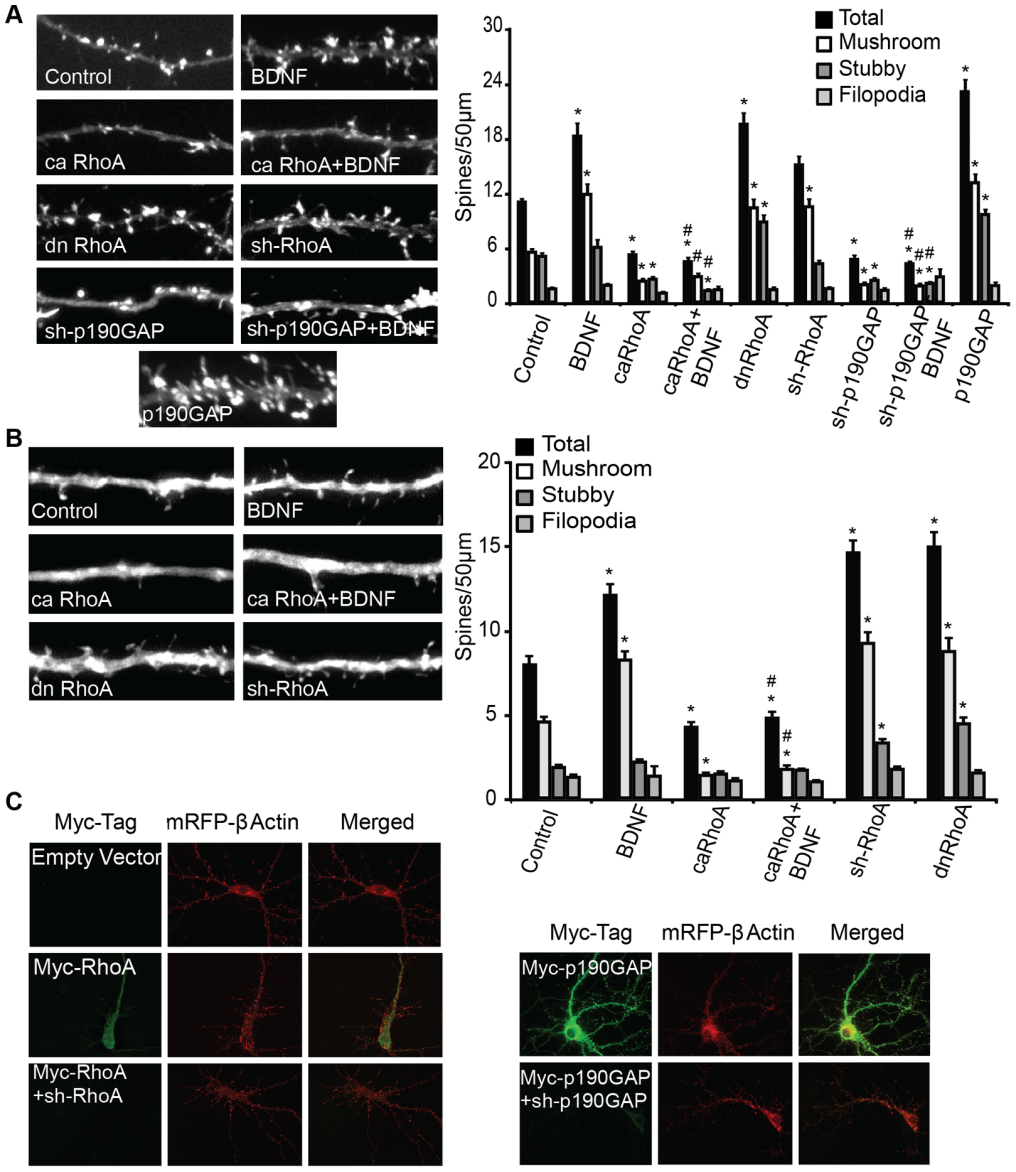
RhoA activity inhibits BDNF-induced dendritic spine formation. A) DIV6 cultured hippocampal neurons were transfected with m-RFP-βActin ± empty vector (Control and BDNF), ± caRhoA, ± dnRhoA, ± sh-RhoA, ± sh-p190GAP, ± p190GAP, then treated ±50 ng/mL BDNF on DIV7 until fixed on DIV12. Dendrites were imaged, and two to three different sections of dendrite per neuron (>24neurons per condition from 2 or more independent experiments were analyzed). Quantification of dendritic spine type and filopodia density is shown, with total spine number representing the combination of mushroom and stubby spines. B) Organotypic hippocampal slice cultures were transfected on DIV2 with Tomato (TFP) (Control and BDNF), ± caRhoA, ± dnRhoA, ± sh-RhoA, then treated ±50 ng/mL BDNF on DIV4 until fixed on DIV6. Dendrites of CA1 pyramidal neurons were imaged, and a single dendrite was analyzed per neuron (20–50 neurons/condition from 3 independent cultures). C) Dissociated hippocampal neurons were transfected on DIV6 with m-RFP-βActin and empty vector, myc-RhoA, myc-RhoA+sh-RhoA, myc-p190GAP, myc-p190GAP+sh-p190GAP. Neurons were fixed on DIV12, and immunostained using anti-myc antibody, and imaged with 60X lens. (± SEM, Statistical analyses utilized ANOVA and Tukey’s post-test, *p<0.001 compared to control, #p<0.001 compared to BDNF).

Many Rho-family proteins play important roles in regulating the actin-cytoskeleton and neuronal morphogenesis. Specifically, the activation of Rac1-mediated signaling is thought to stimulate the formation of dendritic spines, while the activation of RhoA signaling is thought to be inhibitory to spinogenesis [5]–[7], [59]–[62]. Since Par6C and Rnd3 have both been shown to inhibit RhoA signaling, we examined the role of RhoA in BDNF-stimulated synaptogenesis. As in previous studies, BDNF significantly increased dendritic spine density, specifically of mushroom shaped spines (Figure 5A). Expression of constitutively active RhoA, (caRhoA) significantly decreased spine density, and prevented BDNF-induced changes in spine density (Figure 5A). Conversely, expression of dominant negative RhoA, (dnRhoA) or sh-RhoA (Figure 5C) increased dendritic spine density, while having little effect on filopodial projection density (Figure 5A). Additionally, both dnRhoA and sh-RhoA increased average spine head width. Transfection of caRhoA alone did not change average spine head width, but did prevent spine head enlargement following BDNF treatment (data not shown). Par6C and Rnd3 are thought to inhibit RhoA signaling via activation of the RhoAGAP, p190RhoGAP [9]–[14], [23], [45], [63]. As expected, overexpression of p190RhoGAP significantly increased dendritic spine density of all spine types without effecting filopodial density (Figure 5A). Inhibition of p190RhoGAP, using an shRNA construct (Figure 5C), decreased dendritic spine density below control levels, and prevented BDNF-induced increases in spine density (Figure 5A).

Organotypic hippocampal slices retain the cellular and morphological organization of the intact hippocampus and have been used extensively to study activity-dependent synaptic plasticity [16]–[18], [64], [65]. Similar to disassociated cultures, BDNF-treatment promoted an increase in total spine density, except that this increase was restricted to mushroom-shaped spines. Transfection of caRhoA significantly decreased the density of mushroom-shaped spines and blocked BDNF-induced spinogenesis (Figure 5B). dnRhoA and sh-RhoA significantly increased both mushroom and stubby spine density (Figure 5B). Therefore, we conclude that BDNF-induced spine formation involves the inhibition of RhoA, presumably via increased activation of p190GAP.

### Par6C is an Essential Mediator of BDNF-induced Synaptogenesis

**Figure 6:**
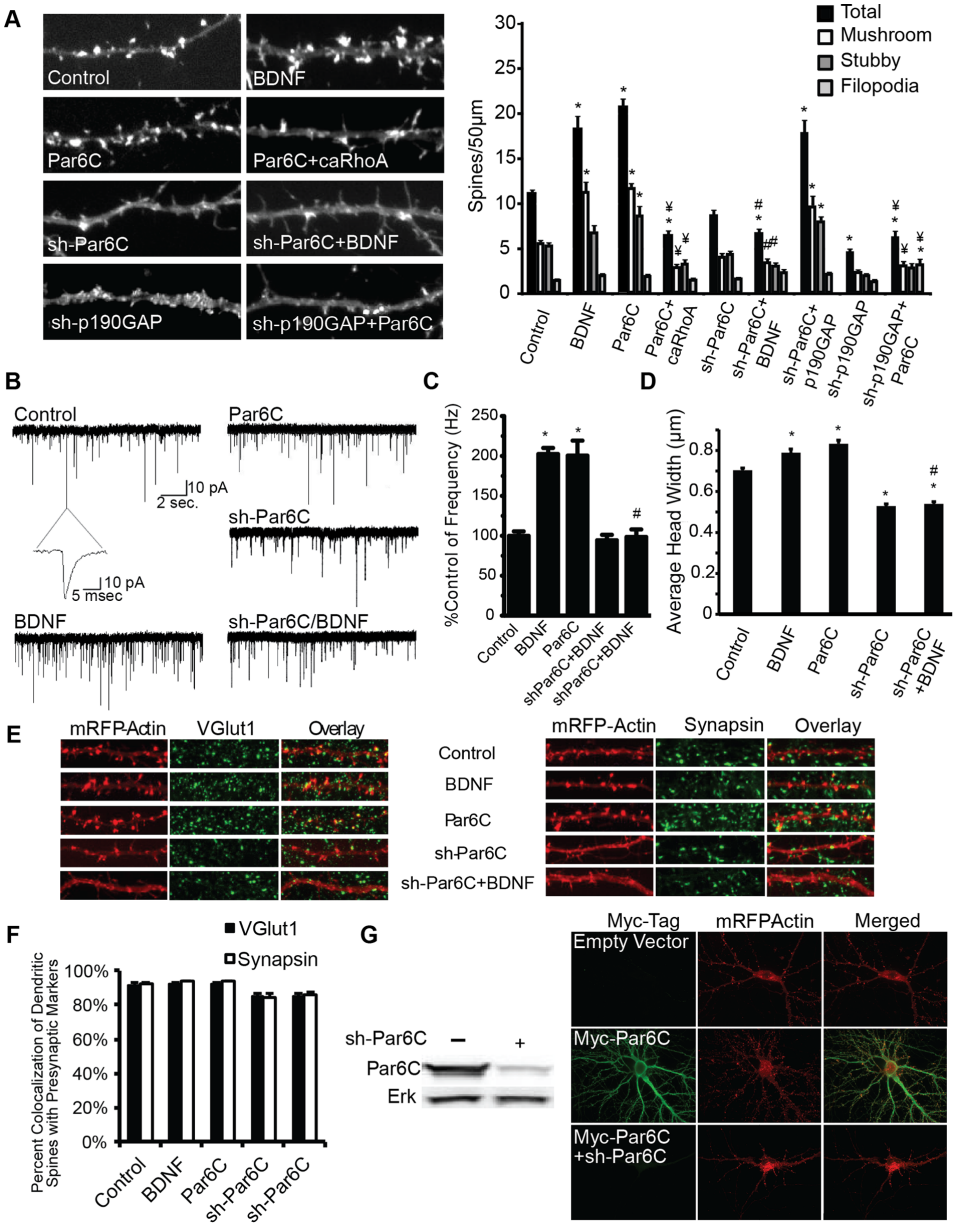
Par6C is an essential mediator of BDNF-induced synaptogenesis. DIV6 cultured hippocampal neurons were transfected with mRFP-βActin ± empty vector (Control and BDNF), ± Par6C, ± Par6C+caRhoA, ± sh-Par6C, ± sh-p190GAP, ± sh-p190GAP+Par6C, ± p190GAP, ± p190GAP+shPart6C, and then treated ±50 ng/mL BDNF on DIV7 until fixed on DIV12. Cultures were then used for electrophysiological recordings, or fixed, mounted, immunostained, and imaged. A) Representative images and quantification of dendritic spine type and filopodia density is shown, with total spine number representing the combination of mushroom and stubby spines (2–3 different dendritic sections (>50 µm) on 24–60 neurons per condition were analyzed in 2 or more experiments). B) Representative traces of mEPSCs recorded from hippocampal neurons. C) Average frequencies of mEPSCs relative to control (20–40 neurons in 2–4 experiments). D) Average spine head width. E) Representative images of neurons immunostained using anti-VGlut1 and anti-Syanapsin1 antibodies. F) Quantification of percent co-localization of presynaptic markers with dendritic spine heads. G) HEK cells transfected with myc-Par6C ± sh-Par6C, and cell lysates analyzed using Western Blot and stained using anti-Par6C and anti-ERK2 antibodies. Representative images of neurons transfected on DIV6 with mRFP-βActin ± empty vector, ± Par6C-myc, or ± Par6C-myc+sh-Par6C. On DIV12 neurons were fixed and immunostained using anti-myc antibody, and imaged with 60X lens. (± SEM, Statistical analyses utilized ANOVA and Tukey’s post-test, *p<0.001 compared to control, #p<0.001 compared to BDNF, ¥p<0.001 compared to Par6C).

Par6C has been shown to stimulate spine formation in hippocampal neurons by controlling p190RhoGAP-mediated inhibition of RhoA [20]–[25]; however, relatively little is known about the mechanisms that regulate Par6C expression and function. Overexpression of Par6C increases the density of both mushroom and stubby spines (Figure 6A). Co-expression of caRhoA with Par6C blocked Par6C-stimulated spinogenesis (Figure 6A). sh-RNA-mediated repression of Par6C expression (Figure 6G) decreased spine densities under basal conditions, and blocked BDNF-induced spinogenesis (Figure 6A). Because, sh-RNA-mediated knockdown can have off target effects we confirmed that expression of an sh-RNA construct targeting a distinct region of the Par6C mRNA transcript also inhibited BDNF-induced spine formation. Additionally, we found that neither a non-scrambled control sh-RNA, nor a sh-RNA that inhibits expression of Rac3 (a RhoGTPase actin regulator not required for spine formation [19], [66]) prevent BDNF-induced increases in dendritic spine density (Figure S1). p190RhoGAP over-expression was effective in stimulating a significant increase in dendritic spine density, and effectively rescued the inhibition of spine formation induced by Par6C suppression (Figure 6A). Furthermore, shRNA-mediated suppression of p190GAP significantly decreased levels of dendritic spine density below basal levels with and without co-expression of Par6C (Figure 6A). Par6C, like BDNF-treatment, significantly increased average spine head width above control levels, while sh-Par6C significantly decreased average spine head width below control levels, with and without BDNF-treatment (Figure 6D). Additionally, both VGlut1 and Synapsin1 were highly co-localized (∼80–90%) with dendritic spines in control, BDNF, and Par6C conditions (Figure 6E and F). Furthermore, expression of Par6C increased the frequency of mEPSCs approximately 2-fold over control levels, close to the levels seen following BDNF stimulation (Figure 6B–C). In contrast, targeted knockdown of Par6C had no significant effect on mEPSC frequency compared to control, but blocked the effect of BDNF on mEPSC frequency. Neither increasing nor decreasing the expression of Par6C affected mEPSC amplitude, rise time, or decay time (data not shown).

### Rnd3 is an Essential Mediator of BDNF-induced Synaptogenesis

**Figure 7:**
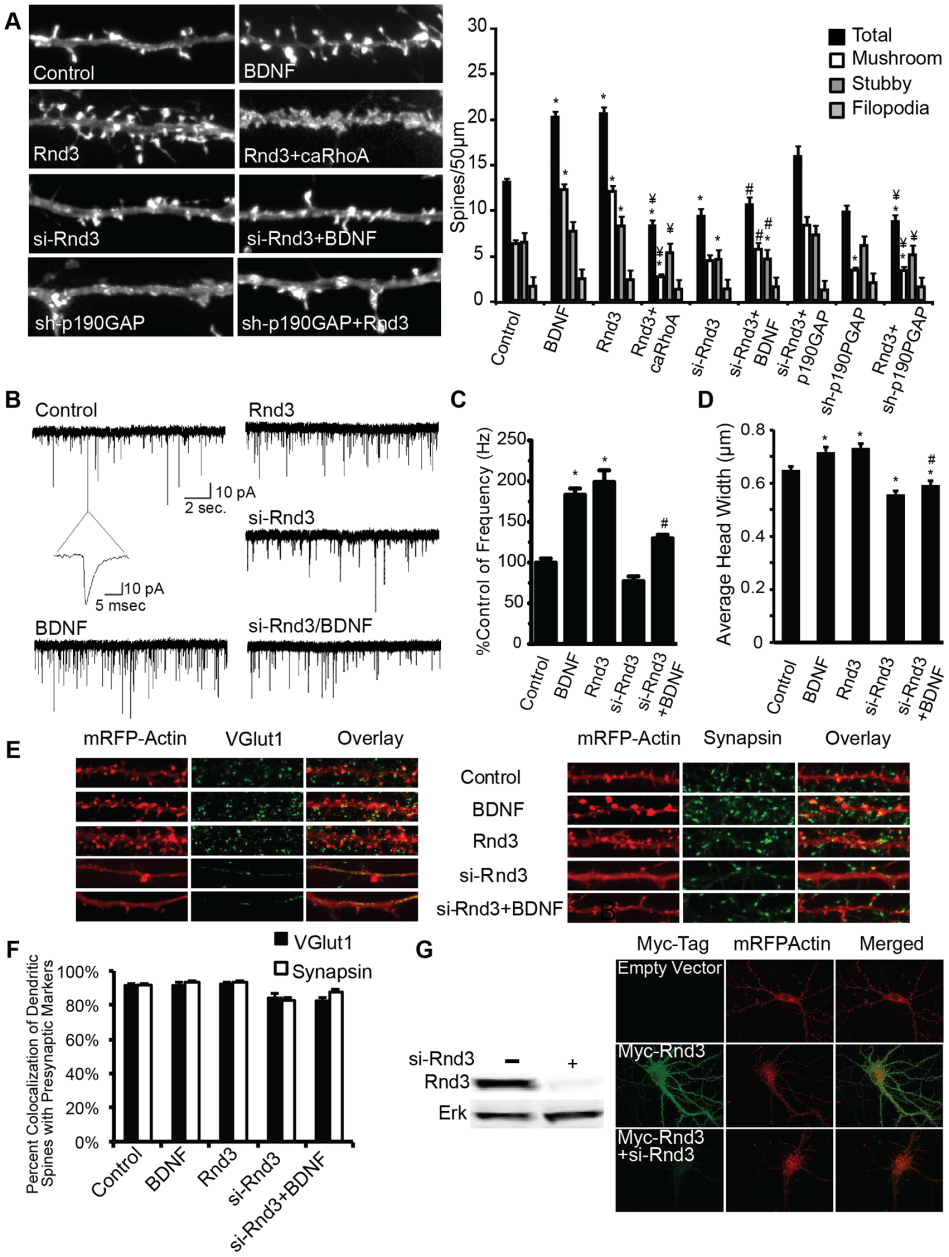
Rnd3 is an essential mediator of BDNF-induced synaptogenesis. DIV6 cultured hippocampal neurons were transfected with m-RFP-βActin ± empty vector (Control and BDNF), ± Rnd3, ± Rnd3+caRhoA, ± si-Rnd3, ± sh-p190GAP, ± sh-p190GAP+Rnd3, ± p190GAP, ± p190GAP+siRnd3 and then treated ±50 ng/mL BDNF on DIV7 until fixed on DIV12. Cultures were then used for electrophysiological recordings, or fixed, mounted, immunostained, and imaged. A) Representative images and quantification of dendritic spine type and filopodia density is shown, with total spine number representing the combination of mushroom and stubby spines (2–3 different dendritic sections (>50 µm) on 24–60 neurons per condition were analyzed in 2 or more experiments). B) Representative traces of mEPSCs recorded from hippocampal neurons. C) Average frequencies of mEPSCs relative to control (20–35 neurons in 2–4 experiments). D) Average spine head width. E) Representative images of neurons immunostained using anti-VGlut1 and anti-Syanapsin1 antibodies. F) Quantification of percent co-localization of presynaptic markers with dendritic spine heads. G) HEK cells transfected with myc-Rnd3± si-Rnd3, and cell lysates analyzed using Western Blot, and stained using anti-Rnd3 and anti-ERK2 antibodies. Representative images of neurons transfected on DIV6 with mRFP βActin ± empty vector, ± myc-Rnd3, or ± myc-Rnd3+si-Rnd3. On DIV12 neurons were fixed and immunostained using anti-myc antibody, and imaged with 60X lens. (± SEM, Statistical analyses utilized ANOVA and Tukey’s post-test, *p<0.001 compared to control, #p<0.001 compared to BDNF, ¥p<0.001 compared to Rnd3).

Rnd3 is a well-established inhibitor of the RhoA signaling pathway that is believed to function by directly activating p190RhoGAP, directly inhibiting the RhoA effector ROCK, and/or inhibiting the RhoA-GEF SYX [27], [45], [48], [67], [68]. Here we show that transfection of neurons with Rnd3 markedly increased dendritic spine density (Figure 7A). Consistent with its role as an inhibitor of RhoA, co-expression of caRhoA with Rnd3 prevented the increase in spine density seen with Rnd3 overexpression alone. Inhibition of Rnd3 expression using siRNA transfection (Figure 7G) reduced dendritic spine density of all spine types (Figure 7A). Moreover, transfection of two distinct si-Rnd3 siRNAs in neurons prevented BDNF-induced increases in spine density (Figure 7A) (Figure S1), suggesting a role for Rnd3 in BDNF-mediated spine formation. Co-transfection of p190RhoGAP with si-Rnd3 increased the density of all spine types significantly above the levels of si-Rnd3 alone; however, these changes were not significant compared to control spine densities and therefore not a complete rescue of Rnd3 inhibition (Figure 7A). On the other hand, sh-RNA-mediated inhibition of p190RhoGAP expression prevented Rnd3-mediated increases in dendritic spine density (Figure 7A). Rnd3 phenocopied the effects of BDNF on average spine head width (Figure 7D). Additionally, si-Rnd3 treatment in the presence or absence of BDNF significantly decreased average spine head width (Figure 7D). Like in Par6C experiments, co-localization of dendritic spines with presynaptic markers was approximately 80–90% in control, BDNF, and Rnd3 conditions (Figure 7E and F). Furthermore, expression of Rnd3 increased the frequency of mEPSCs approximately 2-fold over control, and targeted knockdown of Rnd3 modestly reduced mEPSC frequency, and significantly decreased the effect of BDNF on mEPSC frequency (Figure 7B and C). Neither increasing nor suppressing the expression of Rnd3 affected mEPSC amplitude, rise time or decay time (data not shown). These findings suggest that Rnd3 plays an integral role in BDNF-induced synaptogenesis presumably via inhibition of RhoA.

### Par6C and Rnd3 are Essential Mediators of BDNF-induced Spinogenesis in Organotypic Slice Culture

**Figure 8:**
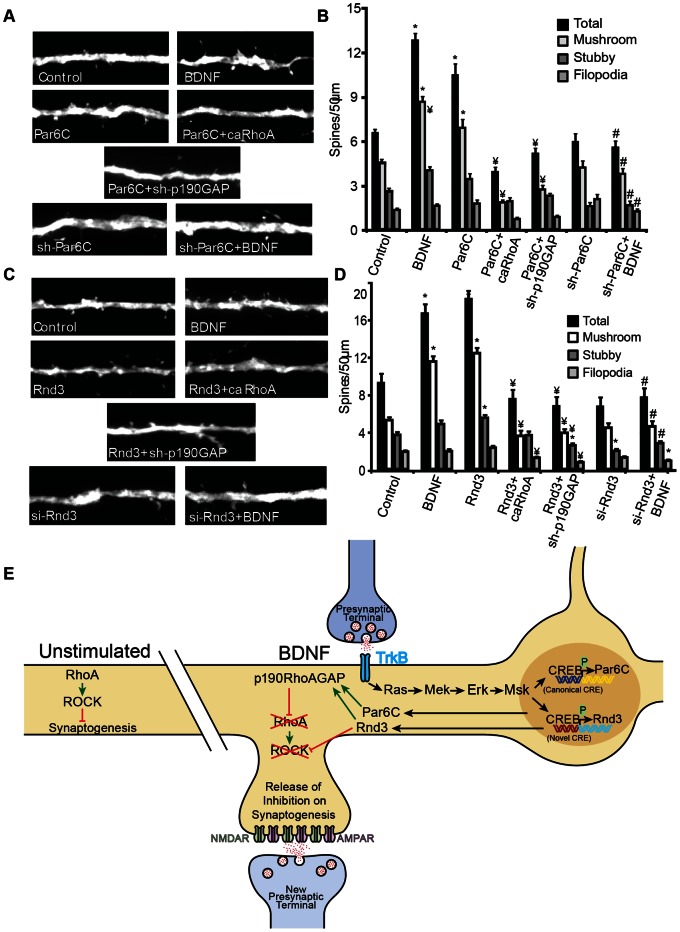
Par6C and Rnd3 are essential mediators of BDNF-induced spinogenesis in organotypic slice cultures. Organotypic hippocampal slice cultures were transfected on DIV2 with Tomato (TFP) (Control and BDNF), A) ± Par6C, ± Par6C+caRhoA, ± Par6C+sh-p190GAP ± sh-Par6C. B) ± Rnd3, ± Rnd3+caRhoA, Rnd3+sh-p190GAP, ± si-Rnd3, and then stimulated with ±50 ng/mL BDNF on DIV4 until fixed on DIV6. Dendrites of CA1 pyramidal neurons were imaged, and a single dendrite was analyzed per neuron (30–60 neurons/condition in 3 separate experiments). (± SEM, Statistical analyses utilized ANOVA and Tukey’s post-test (*p<0.001 compared to control, #p<0.001 compared to BDNF, ¥p<0.001 compared to Par6 or Rnd3). E) Model of BDNF- and CREB-dependent synaptogenesis.

BDNF-treatment increased spine density above control levels, as did over expression of either Par6C or Rnd3 (Figure 8A–B). Co-expression of Par6C or Rnd3 with caRhoA significantly decreased total spine density, specifically that of mushroom spines, below control levels (Figure 8A–B). Furthermore, inhibition of Par6C or Rnd3 using the sh-Par6C or si-Rnd3 constructs had no significant effect on dendritic spine density, but completely prevented BDNF-induced increases in dendritic spines (Figure 8A–B). These data confirm that Par6C and Rnd3 are important mediators of BDNF-induced synaptogenesis.

## Discussion

### Identification of Candidate CREB Target Genes

The identification of genes directly regulated by CREB has largely relied on *in vitro* binding assays and reporter studies. Nevertheless, multiple epigenetic mechanisms, including DNA methylation, histone modifications, and chromatin structure, influence CREB occupancy [25], [29], [69]–[71]. Previous studies of the CREB transcriptional network utilized cells that do not contain mature synapses [31]–[44], [72]. To identify CREB-regulated genes that contribute to synaptogenesis, we generated a ChIP-Seq library from hippocampal neuron chromatin. This led to the identification of ∼33,000 predicted CREB-bound regions. We utilized gene ontology analyses of this ChIP-Seq data set to identify candidate regulators of synaptogenesis and in this study we focused on the RhoA inhibitors, Par6C and Rnd3.

### CREB ChIP-Seq Identifies a Non-canonical CREB Binding Motif

While the Par6C promoter contains a consensus palindromic CRE that overlaps with a CREB ChIP-Seq peak, the Rnd3 ChIP-Seq peak is not adjacent to a canonical CRE. As in earlier studies, CREB ChIP-Seq peaks were significantly enriched for consensus CRE motifs. Nevertheless, as in earlier studies, a significant fraction of peaks did not contain an adjacent CRE. These peaks could represent, noise, indirect interactions with other factors, or a non-canonical CREB-bound motif. Interestingly, *de novo* motif analyses of ChIP-Seq peaks lacking a consensus CRE identified an alternate, non-canonical, motif (TGGCG). This motif was markedly enriched at ChIP-seq peaks (72%), and both the known and non-canonical motifs were detected in (56%) of CREB ChIP-Seq peaks. A high percentage of CREB ChIP-Seq peaks (16%) contained only the non-canonical motif. Both CREB SACO loci [20], [25], [40] and embryonic forebrain CREB ChIP-Seq peaks [23], [43], [45] showed very similar enrichment for the non-canonical motif. Functional assays showed that this motif was sufficient to confer cAMP- and CREB-responsive transcription.

Interestingly, the non-canonical “TGGCG” motif identified in this study was previously described as a cAMP responsive enhancer in the proenkephalin promoter [41], [73], [74]. Although an NF-I family protein was proposed to bind to the proenkephalin TGGCG motif, mutational analyses suggested that a distinct factor conferred cAMP responsiveness [47], [75]. However, the identity of this factor was never resolved. Another set of studies proposed that this motif could enhance transcription via secondary structure base-pairing with an adjacent CRE [48], [76]–[78]. In contrast, our functional genomic, transcriptional, and structural modeling data indicate that this motif can function as a genuine cis-acting CREB regulatory site. Consistent with this idea, both canonical and non-canonical CREs are tightly clustered at the majority of predicted CREB-bound loci in all data sets examined in this study. Interestingly, our analyses show that virtually all well-characterized CREB-regulated immediate response genes contain a cluster of promoter-proximal canonical and non-canonical motifs (Table S2). Our data also show that the non-canonical CRE is sufficient to confer CREB and cAMP responsiveness and helps explain why some CREB bound regions do not contain canonical CREs [40]. In this manuscript we describe 6,515 additional predicted CREB target genes that contain only a promoter-proximal non-canonical motif, including, Pdgfb, Bmp7, Trk3, Vamp4, Whsc2, and Faf1. Interestingly, mutation of the Whsc2 gene is associated with mental retardation and Faf1 is a Fas-related regulator of apoptosis.

### CREB-dependent Transcription is Essential to BDNF-induced Synaptogenesis

CREB activation has been shown to stimulate the formation of dendritic spines and new synapses [25], [29], [30], [53], [54], [79]. Additionally, neurotrophins, such as BDNF, have been shown to stimulate dendritic spines (at least partially) via a CREB-dependent mechanism [20], [27], [31], [54]. BDNF triggers the rapid phosphorylation of CREB Ser133, and is believed to be an important regulator of CREB-dependent transcription in the nervous system [36], [40], [80]. The proposed signaling pathway linking BDNF to CREB-dependent transcription has been extensively researched, wherein BDNF-treatment potently activates MEK-ERK pathways, and the downstream kinases Msk1/2 and Rsk2 [41], [56], [81], [82] (Figure 8). Although multiple pathways have been proposed, deletion or knockdown of Msk1/2 in neurons almost completely attenuates BDNF-stimulated Ser133 phosphorylation and markedly decreases transcription of CREB-regulated genes [40], [41]. Furthermore, the activity regulated kinase, CaMKI is also an upstream activator of the MEK-ERK pathway suggesting crosstalk between these two pathways at the level of CREB activation [54], [56].

### Identification of CREB-induced Mediators of Synaptogenesis

Although both BDNF- and CREB-dependent transcription are known to stimulate synaptogenesis, the precise mechanism and molecular changes involved are not well characterized. We show that inhibition of RhoA activity is necessary for BDNF-induced spinogenesis in hippocampal neurons. Bioinformatic analysis of CREB ChIP-seq data identified two known inhibitors of Rho, Par6C and Rnd3. We showed that Par6C expression is induced by BDNF in a CREB-dependent fashion, and that Par6C expression is necessary for BDNF-induced synaptogenesis. Similarly, Rnd3 was also transcriptionally regulated by BDNF in a CREB-dependent manner. Unexpectedly, we found that CREB bound to a non-canonical CRE in the Rnd3 promoter region. Moreover, we show here that Rnd3 expression is necessary and sufficient for synaptogenesis in hippocampal neurons. Interestingly, Rnd3 knockout mice are phenotypically similar to BDNF knockout mice, in that Rnd3 deletion leads to retarded growth, postnatal lethality, and severe motor disruption [57], [83]. In future studies, it would be interesting to examine the role of BDNF, Par6C, and Rnd3 in adult or developmental synaptic plasticity.

This study utilized high-throughput ChIP-Seq to identify ∼30,000 predicted CREB target sites not detected in other studies. Moreover, we have characterized and demonstrated the functionality of a non-canonical CRE motif that is enriched to approximately the same extent as the canonical motif in multiple ChIP-Sequencing data sets. Lastly, we have demonstrated how targeted analysis of the CREB ChiP-Seq data in this study can be used to identify CREB-regulated genes that may regulate a wide variety of neuronal processes.

## Supporting Information

Figure S1 **RNAi control experiments.** DIV6 cultured hippocampal neurons were transfected with mRFP-βActin ± empty vector (Control and BDNF), ± sh-Scrambled (non-specific sh-RNA construct), ± sh-Rac3 (sh-RNA construct targeting actin regulator that does not effect spine formation ([1], supplemental figure 6), ± sh-Par6C Alt (Alternate sh-RNA that targets a different region of Par6C transcript), ± si-Rnd3 Alt (Alternate si-RNA sequence targeting different region of Rnd3 transcript), and then treated ±50 ng/mL BDNF on DIV7 until fixed on DIV12. Representative images and quantification of dendritic spine type and filopodia density is shown, with total spine number representing the combination of mushroom and stubby spines (2–3 different dendritic sections >50 µm on 6–24 neurons per condition were analyzed in two experiments). B) Representative images of neurons transfected on DIV6 with mRFP-βActin, ± myc-Par6C, ± myc-Par6C+sh-Par6C Alt, ± myc-Par6C+sh-Scrambled, ± myc-Rnd3, ± myc-Rnd3+si-Rnd3 Alt, ± myc-Rnd3+sh-Scrambled. On DIV13 neurons were fixed and immunostained using anti-myc antibody, and imaged with 60X lens (± SEM, Statistical analyses utilized ANOVA and Tukey’s post-test, *p<0.05 compared to control, #p<0.05 compared to BDNF).
(TIF)

Table S1 Primers used in current study.
(XLS)

Table S2 Annotation of hippocampal neuron CREB ChIP-Seq data with canonical (TGACG), novel (TGGCG) CRE motifs, and closest RefSeq gene transcriptional start site (5′ end; UCSC Genome Browser, mm9). Both strands were used for motif analyses.
(XLS)

Table S3 Annotation of hippocampal neuron CREB ChIP-Seq data with closest embryonic forebrain CREB ChIP-Seq data (re-analysis of [2],closest CREB PC12 ChIP-SACO locus (mapped to UCSC genome browser mm9, [3] and closest RefSeq gene transcriptional start site (5′ end; UCSC Genome Browser, mm9).
(XLS)

Text S1 Supporting Information Legend.
(DOCX)
