# Supplementary figures and images for: A Genome-Wide Screen of CREB Occupancy Identifies the RhoA Inhibitors Par6C and Rnd3 as Regulators of BDNF-Induced Synaptogenesis
Source: PLoS One. 2013 Jun 6;8(6):e64658. doi: 10.1371/journal.pone.0064658 (PMC3675129; doi:10.1371/journal.pone.0064658)

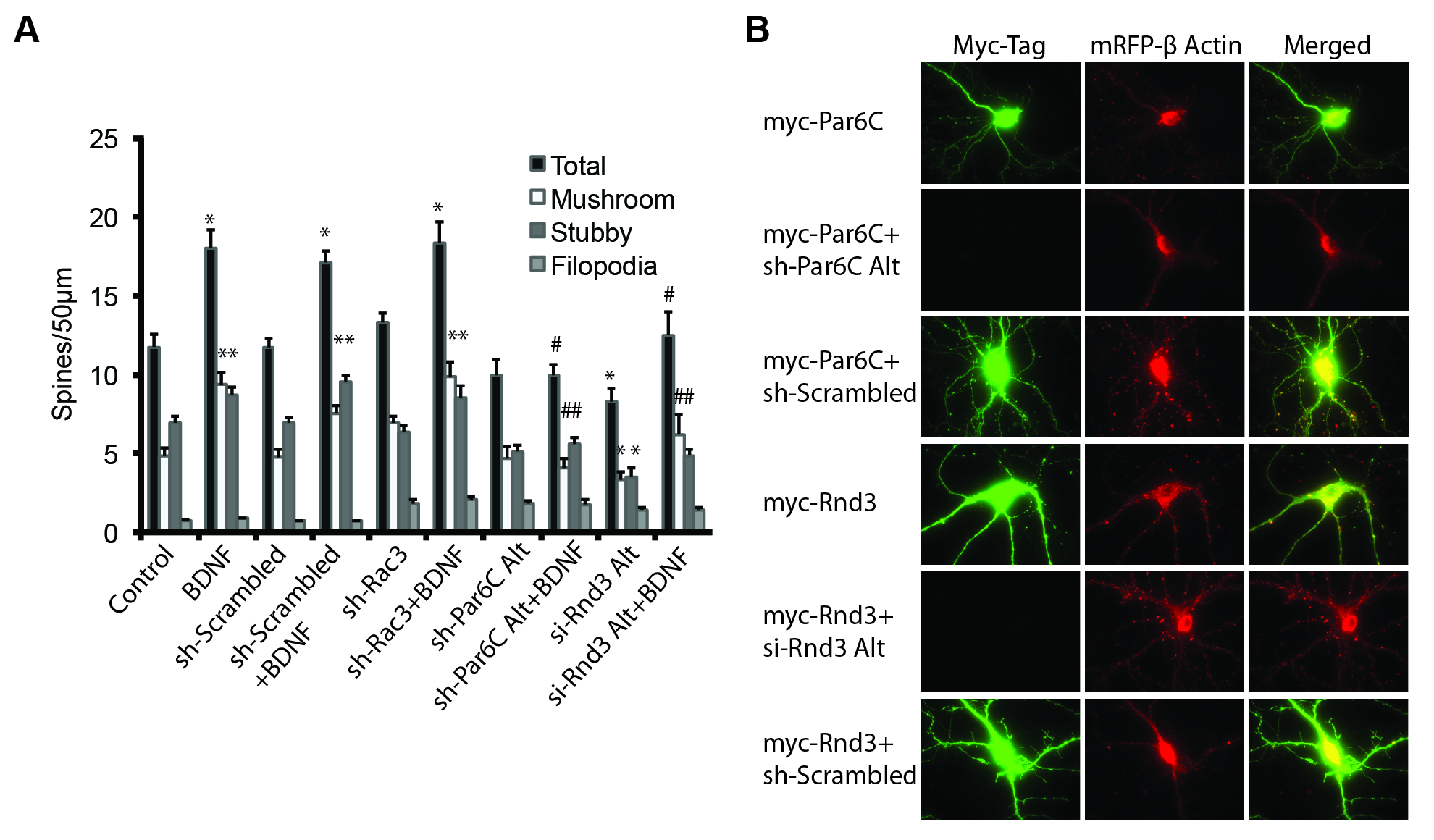

Supplement: Figure S1 — RNAi control experiments. DIV6 cultured hippocampal neurons were transfected with mRFP-βActin ± empty vector (Control and BDNF), ± sh-Scrambled (non-specific sh-RNA construct), ± sh-Rac3 (sh-RNA construct targeting actin regulator that does not effect spine formation ([1], supplemental figure 6), ± sh-Par6C Alt (Alternate sh-RNA that targets a different region of Par6C transcript), ± si-Rnd3 Alt (Alternate si-RNA sequence targeting different region of Rnd3 transcript), and then treated ±50 ng/mL BDNF on DIV7 until fixed on DIV12. Representative images and quantification of dendritic spine type and filopodia density is shown, with total spine number representing the combination of mushroom and stubby spines (2–3 different dendritic sections >50 µm on 6–24 neurons per condition were analyzed in two experiments). B) Representative images of neurons transfected on DIV6 with mRFP-βActin, ± myc-Par6C, ± myc-Par6C+sh-Par6C Alt, ± myc-Par6C+sh-Scrambled, ± myc-Rnd3, ± myc-Rnd3+si-Rnd3 Alt, ± myc-Rnd3+sh-Scrambled. On DIV13 neurons were fixed and immunostained using anti-myc antibody, and imaged with 60X lens (± SEM, Statistical analyses utilized ANOVA and Tukey’s post-test, *p<0.05 compared to control, #p<0.05 compared to BDNF). (TIF) [file pone.0064658.s001.tif]
