# Supplementary material for: A Genome-Wide Screen of CREB Occupancy Identifies the RhoA Inhibitors Par6C and Rnd3 as Regulators of BDNF-Induced Synaptogenesis
Source: PLoS One. 2013 Jun 6;8(6):e64658. doi: 10.1371/journal.pone.0064658 (PMC3675129; doi:10.1371/journal.pone.0064658)
Supplement: Text S1 — Supporting Information Legend. (DOCX) [file pone.0064658.s005.docx]

1. Impey S, Davare M, Lesiak A, Fortin D, Ando H, et al. (2010) An activity-induced microRNA controls dendritic spine formation by regulating Rac1-PAK signaling. Molecular and Cellular Neuroscience 43: 146–156. doi:10.1016/j.mcn.2009.10.005.
2. Kim AH, Reimers M, Maher B, Williamson V, McMichael O, et al. (2010) MicroRNA expression profiling in the prefrontal cortex of individuals affected with schizophrenia and bipolar disorders. Schizophr Res 124: 183–191. doi:10.1016/j.schres.2010.07.002.

3. Impey S, McCorkle SR, Cha-Molstad H, Dwyer JM, Yochum GS, et al. (2004) Defining the CREB Regulon A Genome-Wide Analysis of Transcription Factor Regulatory Regions. Cell 119: 1041–1054. doi:10.1016/j.cell.2004.10.032.
